# 

**Published:** 1885-06

**Authors:** 


### OUR ADVERTISERS.

BRADFIELD & WARE, Atlanta.—Mr. L. H. Bradfield, the well-known druggist on Whitehall, has sold a half interest in his store to Mr. W. R. Ware. Mr. Ware is a thorough pharmacist, and will add much to this already popular house.

BUFFALO LITHIA WATER.—Of this justly popular water, advertised on the first page of THE JOURNAL, Dr. Robert Battey, of Rome, says: "I would state that I have been using the Buffalo Lithia Water, No. 2, in my practice for three years past in cases of chronic inflammation of the bladder, whether induced by stone, by enlarged prostate in the aged, or by neglected gonorrhœa, and have secured excellent results, which encourages me to prescribe it for the future."

DAVID NICHOLSON, St. Louis.—See advertisement of this gentleman's "Liquid Bread," to be found on page 35. It is one of the most pleasant preparations of malt we know.

RIO CHEMICAL CO., St. Louis.—Dr. W. T. Leachman, of Louisville, writes of Celerina, one of the preparations of this Company: "I have used Celerina in the treatment of nervous diseases with the most *gratifying* results, and in a few cases of *opium* habit. I am thoroughly satisfied with its remedial effects in this particular affliction."

FELLOW'S HYPOPHOSPHITES.—This preparation contains the essential elements to the animal organism. It is largely used in the treatment of the affections of the respiratory organs and in nervous troubles. See advertisement, and write for samples and descriptive circulars, which will be sent free.

BATTLE & CO., St. Louis.—See advertisement of Papine on page 2. Of this preparation Dr. Geo. H. H. Williams, of Philadelphia, says: "From a somewhat extended experience in prescribing Papine, I am led to regard it as the safest and best of all the opiates."

